# Unexpected Self‐Assembly of Nanographene Oxide Membranes upon Electron Beam Irradiation for Ultrafast Ion Sieving

**DOI:** 10.1002/advs.202404001

**Published:** 2024-07-08

**Authors:** Fangfang Dai, Zonglin Gu, Shouyuan Hu, Bingquan Peng, Rujie Yang, Jie Jiang, Lufeng Yao, Shanshan Liang, Yusong Tu, Pei Li, Liang Chen

**Affiliations:** ^1^ School of Physical Science and Technology Ningbo University Ningbo 315211 China; ^2^ Wenzhou Institute University of Chinese Academy of Sciences Wenzhou Zhejiang 325000 China; ^3^ School of Physical Science and Technology & Microelectronics Industry Research Institute Yangzhou University Jiangsu 225009 China; ^4^ Department of Physics East China University of Science and Technology Shanghai 200237 China; ^5^ Department of Basic Courses Naval University of Engineering Wuhan 430033 China; ^6^ State Key Laboratory of Surface Physics and Department of Physics Fudan University Shanghai 200433 China

**Keywords:** electron beam irradiation, nanographene oxide (nGO) membrane, ultrafast ion sieving, ultrahigh water permeance

## Abstract

Nanographene oxide (nGO) flakes—graphene oxide with a lateral size of ≈100 nm or less—hold great promise for superior flux and energy‐efficient nanofiltration membranes for desalination and precise ionic sieving owing to their unique high‐density water channels with less tortuousness. However, their potential usage is currently limited by several challenges, including the tricky self‐assembly of nano‐sized flakes on substrates with micron‐sized pores, severe swelling in aqueous solutions, and mechanical instability. Herein, the successful fabrication of a robust membrane stacked with nGO flakes on a substrate with a pore size of 0.22 µm by vacuum filtration is reported. This membrane achieved an unprecedented water permeance above 819.1 LMH bar^−1^, with a high rejection rate of 99.7% for multivalent metal ions. The nGO flakes prepared using an electron beam irradiation method, have uniquely pure hydroxyl groups and abundant aromatic regions. The calculations revealed the strong hydrogen bonds between two nGO flakes, which arise from hydroxyl groups, coupled with hydrophobic aromatic regions, greatly enhance the stability of stacked flakes in aqueous solutions and increase their effective lateral size. The research presents a simple yet effective approach toward the fabrication of advanced 2D nanographene membranes with superior performance for ion sieving applications.

## Graphical abstract

A simple yet effective approach toward the fabrication of nanographene oxide (nGO) membranes with superior performance for ion sieving is developed in this work.

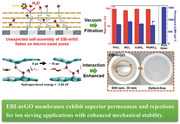

## 1 Introduction

2D graphene oxide (GO) membranes have attracted substantial interest for their precise ionic sieving, with applications in water desalination and purification,^[^ ^1^, ^2^, ^3^, ^4^, ^5^, ^6^ ^]^ gas and ion separation,^[^ ^7^, ^8^, ^9^, ^10^ ^]^ biosensors,^[^ ^11^ ^]^ proton conductors,^[^ ^12^ ^]^ lithium‐based batteries^[^ ^13^ ^]^ and super‐capacitors.^[^ ^14^ ^]^ Both an ultrahigh water permeance and capability of precise sieving of GO membranes^[^ ^15^, ^16^, ^17^, ^18^ ^]^ are crucial for their extensive future filtration applications. In particular, nanographene oxide (nGO) flakes, one of the simplest and most widely studied small GO flakes are thought to have potential for high‐flux and energy‐efficient membranes because of their shorter and less tortuous water channels,^[^ ^19^, ^20^, ^21^, ^22^ ^]^ especially those with a lateral size of ≈100 nm. However, several challenges need to be overcome to achieve small flake GO membranes with desirable high performance, including the difficult self‐assembly of nano‐sized flakes on most substrates with micron‐sized pores,^[^ ^19^ ^]^ severe swelling in aqueous solution,^[^ ^23^ ^]^ and mechanical instability.^[^ ^24^ ^]^

Numerous efforts have been devoted to producing stable membranes stacked with small GO flakes on substrates with micron‐sized pores. For example, the membrane can be stably assembled from small reduced‐GO (rGO) flakes (with a lateral size of 0.1–1 µm)^[^ ^20^ ^]^ enhanced by cationic cross‐linking^[^ ^19^, ^25^ ^]^ with robust stability, and reach an average value of ≈100 L m^−2^ h^−1^ bar^−1^ (LMH bar^−1^) for various dye rejection applications. Besides, other nanoparticles and long‐chain cross‐linkers^[^ ^26^, ^27^ ^]^ are also used to construct stabilized membranes, including tiny GO nanosheets layered on a microporous membrane substrate. Despite great improvements, however, stacking the smaller nanographene flakes (close to or less than 100 nm) stably to exclude small cations with desirable ultrahigh water permeance remains difficult.

Here, we achieved a robust membrane stacked with nGO flakes with an average lateral size of ≈100 nm on a substrate with a pore size of 0.22 µm by vacuum filtration. The nGO flakes had relatively pure hydroxyl groups and a significant number of aromatic regions, which were obtained from the initial GO suspension via electron beam irradiation assisted by amino‐hydrothermal reduction (EBI‐nrGO). Ab initio molecular dynamics simulations (AIMD) revealed that the strong hydrogen bond between the hydroxyl groups in two neighboring EBI‐nrGO flakes formed a stable connection, robustly locking the interlayer of the two flakes in aqueous solution. Relatively pure hydroxyl groups on the EBI‐nrGO flakes obtained by the selective reduction of epoxy groups weakened the hydrogen‐bond network in the plane, together with the hydrophobic aromatic regions, greatly improving the stability of the stacked flakes. Importantly, the EBI‐nrGO membrane achieved unprecedented water permeance with a high rejection rate for multivalent metal ions (Fe^3+^, Al^3+^, Cu^2+^, and Pb^2+^). Notably, this is the most permeable membrane with a satisfactory rejection rate for multivalent ions among all state‐of‐the‐art nano‐filtration membranes. Moreover, the EBI‐nrGO membrane exhibited mechanical and sieving stability during the long‐term operation.

## 2 Results and Discussion

### 2.1 Fabrications and Characterizations of the EBI‐nrGO Membranes

**Figure 1:**
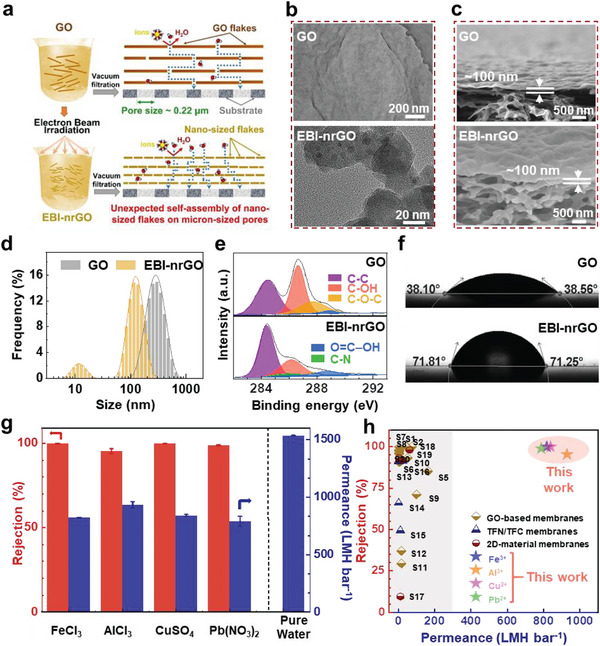
The unprecedented water permeances and high rejection rates of the EBI‐nrGO membranes. a) Schematic of the fabrications and transport channels of the GO and EBI‐nrGO membranes. b) The TEM images of the GO and EBI‐nrGO flakes. c) The cross‐sectional SEM images of the GO and EBI‐nrGO membranes. d) The size distributions of the GO and EBI‐nrGO flakes measured by nano particle size analyzer. e) The XPS spectra of C 1s for the GO and EBI‐nrGO membranes. f) The water contact angles (WCAs) of the GO and EBI‐nrGO. g) The water permeances (blue bars) and rejection rates (red bars) of the EBI‐nrGO membranes at a thickness of ≈100 nm for different multivalent ions and pure water. The error bars are the statistical standard deviations calculated from three independent experiments with three different membranes. h) Comparison of the water permeance (x‐axis) and salt rejection (y‐axis) of the EBI‐nrGO membrane among the state‐of‐the‐art nanofiltration membranes reported in the literature for multivalent ion removal (dark yellow quadrilateral, GO‐based membranes; dark blue triangle, TFN/TFC membranes; dark red circle, other 2D material membranes; details in Section S1; Supporting Information).

The EBI‐nrGO suspension was prepared from the GO suspension using electron beam irradiation and the amino‐hydrothermal method (see details in the Experimental sections). The size of the EBI‐nrGO flakes ranged from 50 to 120 nm with a few measuring ≈10 nm and an average size of ≈100 nm, which is much smaller than that of the untreated GO with a size of 100–1000 nm (**Figure** **1**b,d). X‐ray photoelectron spectroscopy (XPS) C1s spectra showed that the EBI‐nrGO had a lower oxygen content with 26.6% hydroxyl groups and negligible amounts of other oxygen‐containing groups (<5%), while the untreated GO has higher hydroxyl groups (38.4%) and epoxy groups (17.2%) (Figure 1e)^[^ ^28^ ^]^ As shown in Figure 1a, GO and EBI‐nrGO membranes were prepared from 10 mL and 12.5 mg L^−1^ suspensions on mixed cellulose ester (MCE) substrates with a pore size of 0.22 µm using vacuum filtration, respectively. Scanning electron microscopy (SEM) images showed that the obtained two membranes had a thickness of ≈100 nm, were continuous and were free of macro‐pores or defects (Figure 1c; Figure S10, Supporting Information). The water contact angles (WCAs) on the GO and EBI‐nrGO membranes were ≈38.3° and 71.5° respectively (Figure 1f), indicating that the EBI‐nrGO membrane had an enhanced hydrophobicity.

### 2.2 Ultrafast Ion Sieving Performance of the EBI‐nrGO Membranes

The EBI‐nrGO membrane exhibited unprecedented performance in multivalent metal ion rejection. Different multivalent metal ions (50 mg L^−1^ each; FeCl_3_, AlCl_3_, CuSO_4_, and Pb(NO_3_)_2_) were added to the feed side of the dead‐end filtration set‐up. At a pressure of 1 bar, the salt solutions were filtered through the EBI‐nrGO membranes (see details in the Experimental Sections: S2 and S3; Supporting Information). As shown in Figure 1g, the water permeance of the EBI‐nrGO membranes reached up to 819.1 ± 4.8, 930.9± 27.7, 839.8 ± 7.9, and 788.3 ± 43.0 LMH bar^−1^ for FeCl_3_, AlCl_3_, CuSO_4_, and Pb(NO_3_)_2_, with corresponding rejection rates of 99.7 ± 0.2%, 95.2 ± 1.5%, 99.7 ± 0.1%, and 98.7 ± 0.3%, respectively. Statistical analyses were based on the data from three parallel samples in each group and every sample contributed three data points. In contrast, the permeance values of the GO membranes were ≈47.3 ± 7.1 LMH bar^−1^ with a rejection rate of 99.2 ± 0.1% for FeCl_3_ (Figure S2, Supporting Information). Thus, we achieved a more than twenty‐fold increase in water permeance through EBI‐nrGO membranes. Notably, the ultrafast ion sieving of the EBI‐nrGO membranes is far superior to the highest permeance of all state‐of‐the‐art nanofiltration membranes for multivalent metal‐ion solutions (Figure 1h), as the highest permeance reported was only 164.7 LMH bar^−1^ with a moderate ion rejection rate of 85.2%.^[^ ^29^ ^]^

**Figure 2:**
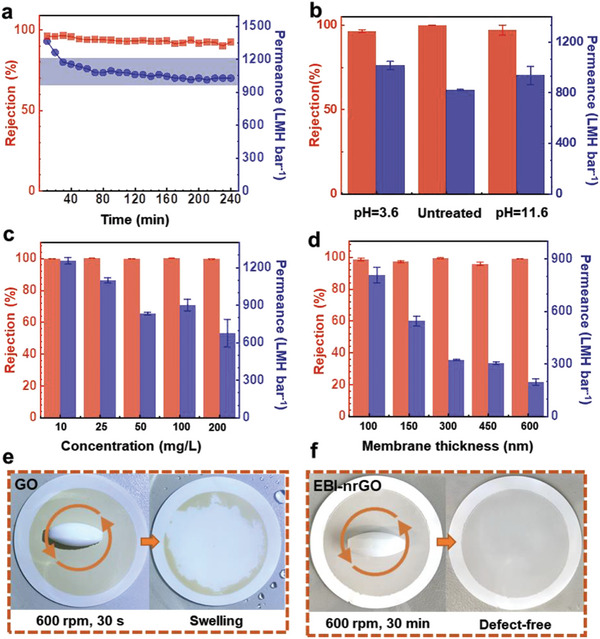
The ion sieving and mechanical stabilities of the EBI‐nrGO membranes. a) The long‐term operation stability test on EBI‐nrGO membranes (50 mg L^−1^ FeCl_3_ solution, membrane thickness of ≈100 nm). b) The acid‐and‐alkaline environment test on the EBI‐nrGO membranes (50 mg L^−1^ FeCl_3_ solution; membrane thickness of ≈100 nm). c) The concentration effect on the EBI‐nrGO membranes (FeCl_3_ solutions; membrane thickness of ≈100 nm). d) The performance of the EBI‐nrGO membranes at different thicknesses (50 mg L^−1^ FeCl_3_ solution). e) The mechanical property of the GO membrane in aqueous solution. A magnetic rotor was placed on the membrane surface, rotating at 600 rpm for 30 s. f) The mechanical property of the EBI‐nrGO membrane in aqueous solution. A magnetic rotor was placed on the membrane surface, rotating at 600 rpm for 30 min. The error bars are the statistical standard deviations calculated from three independent experiments with three different membranes.

The long‐term operation of the EBI‐nrGO membranes exhibited excellent robustness and durable stability. We prepared 1000 mL of a 50 mg L^−1^ FeCl_3_ solution in a beaker, and then transported the solution to the feed side with a peristaltic pump, while maintaining 300 mL of solution on the feed side, to form a cyclic flow between the feed side and the beaker, similar to the cross‐flow method.^[^ ^17^, ^30^, ^31^ ^]^ As shown in **Figure** **2**a, the water permeance decreased slightly from 1362.2 to 1026.6 LMH bar^−1^ for 240 min, while the corresponding rejection rate was maintained at more than 90.0% for the FeCl_3_ solution. The slight decrease in water permeance was mainly due to the improved compact structures between the GO flakes under long‐term vacuum filtration conditions.^[^ ^21^, ^32^ ^]^ Notably, during long‐term operation, the salt concentration on the membrane surface continued to increase, thus affecting the performance of the membrane. This type of cross‐flow device can improve the concentration enrichment on the membrane surface, but the rejection rate still decreased slightly.^[^ ^17^ ^]^ SEM images (Figure 1c) show that the two membranes obtained had a thickness of ≈100 nm and were continuous and free of macro‐pores or defects, which is critical for a highly efficient separation process because the uniform membrane surface permits water flux with low flow resistance and provides a good environment^[^ ^33^ ^]^ for the interactions of GO flakes with themselves or with ions. In addition, organic fouling, high‐pressure filtration experiments, and acid‐and‐alkaline environment tests were performed to test the stability of the EBI‐nrGO membranes (Figure 2b; Sections S5 and S6; Supporting Information). These results clearly demonstrated the outstanding stability with superior filtration performance of the EBI‐nrGO membranes.

The effect of salt concentration (Figure 2c) and membrane thickness (Figure 2d) on the ion sieving performance was studied. Increasing the salt concentration from 10 to 200 mg L^−1^, clearly reduced in the water permeance from 1253.2 to 675.6 LMH bar^−1^ for the EBI‐nrGO membrane with a thickness of 100 nm, with the corresponding rejection rates of 99.9–99.6%. Increasing the membrane thickness from 100 to 600 nm reduced the water permeance from 806.5 to 196.5 LMH bar^−1^ that shows a negative relationship between the two. These results are consistent with the water flow in the GO channels described by the Hagen–Poiseuille equation,^[^ ^34^, ^35^ ^]^ indicating that the 2D water channel characteristics of our membranes self‐assemble from nano‐sized flakes. Notably, the high rejection rates and stability of the EBI‐nrGO membranes in both the long‐term operation and salt gradient experiments illustrate that the rejection performance benefits from rejection instead of adsorption effects. The adsorption capacities reached a significant value (12–60 g g^−1^; Section S13; Supporting Information), which was much higher than that (0.17 g g^−1^) in our previous work,^[^ ^36^ ^]^ if it was assumed that the removal of ions from the filtrates in our filtration experiments was completely explained by adsorption. Additionally, data from the filtration experiments (Figure 2a,c,d; Figure S9; Supporting Information) exhibited no adsorption saturation characteristics, as the rejection rates dropped sharply to near zero. Therefore, the effect of adsorption was neglected in this study.

In addition, the EBI‐nrGO membrane exhibited superior mechanical property, which is an important factor affecting the functional performance and application scenarios of materials.^[^ ^37^, ^38^ ^]^ A magnetic rotor was placed on the membranes and stirred at 600 rpm to form a cross‐flow in the aqueous solution, as shown in Figure 2e,f (Section S7; Supporting Information). The surface of the GO membrane swelled and broke within 30 s, whereas the EBI‐nrGO membrane remained defect‐free even after 30 min, demonstrating good mechanical stability during filtration.

### 2.3 Ab Initio Molecular Dynamics (AIMD) Simulations

**Figure 3:**
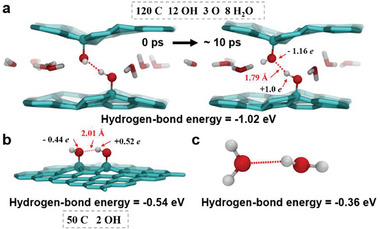
Ab initio molecular dynamics simulations of the EBI‐nrGO flakes. a) Configurations of the hydrogen bond formed between two hydroxyl groups in two neighboring EBI‐nrGO flakes at 0 ps (left) and ≈10 ps (right). b) The hydrogen bond formed between two hydroxyl groups in the same plane of EBI‐nrGO flake. c) The hydrogen bond formed between two water molecules. Carbon, oxygen, and hydrogen atoms are showed in cyan, red, and white, respectively. Hydrogen bonds are illustrated with red dotted lines. The dashed grey texts give the numbers of carbons, hydroxyl groups, oxygen atoms and water molecules contained in the calculation models.

To illustrate the underlying physical mechanism of these membranes, AIMD simulations were performed (more details are provided in the Experimental Sections). Calculations revealed that the hydrogen‐bond energy between the hydroxyl groups in two neighboring EBI‐nrGO flakes was −1.02 eV (**Figure** **3**a), much stronger than those between the hydroxyl groups in the same flake (−0.54 eV) (Figure 3b) or between two water molecules in liquid water (−0.36 eV) (Figure 3c). During the simulation with a duration of 10 ps (Figure 3a), the hydrogen bonds between two neighboring EBI‐nrGO flakes remained very stable, featuring a formation duration much longer than the lifetime of hydrogen bonds within water molecules in liquid water (0.5 ps).^[^ ^25^ ^]^ In contrast, the hydrogen‐bond energy shown in Figure 3c was calculated based on a typical water pair in liquid water, as demonstrated in a previous work.^[^ ^39^ ^]^

To further elucidate this mechanism, we present the atomic charge distributions of the hydrogen atoms in two types of the hydrogen bonds: in the same flake (Figure 3b) and in two neighboring flakes (Figure 3a). As shown, the charge of the hydrogen atom in Figure 3a is approximately twice that of the hydrogen atom in Figure 3b, and the hydrogen‐bond length (1.79 Å) in Figure 3a is shorter than that in Figure 3b (2.01 Å), which explains the stronger hydrogen bond in two neighboring flakes compared to that in the same flake. Therefore, the strong hydrogen bonds formed by the two hydroxyl groups in the two neighboring EBI‐nrGO flakes can easily regulate the interfacial interaction between these two planes, by which the interlayer spacing (Figure S6, Supporting Information) could also be controlled.

EBI‐nrGO had a low oxygen content of hydroxyl groups and negligible epoxy groups, compared to the initial GO with both high hydroxyl and epoxy groups (Figure 1e). Notably, the epoxy groups had little effect on the solubility of the solutes in water^[^ ^40^ ^]^ because of the formation of hydrogen bonds between the epoxy and hydroxyl groups in the plane of the GO flake. The hydrogen‐bond energies in two GO flakes with high oxygen content and two hydroxylated EBI‐nrGO flakes were −0.69 and −0.91 eV (Section S8; Supporting Information), respectively, significantly lower than that between hydroxyl groups in two flakes with only one hydrogen bond (−1.02 eV in Figure 3a), implying that there are much stronger interactions between the hydroxyl groups of the EBI‐nrGO flakes and that a low hydroxyl groups content can enhance these interactions. The selective reduction of the epoxy groups can weaken the hydrogen‐bond network in the plane, strengthen the network between the hydroxyl groups of the EBI‐nrGO flakes, and increase the number of hydrophobic aromatic regions, thereby significantly improving the stability of the stacked flakes in aqueous solutions. Such stable hydrogen bonds, along with the large number of frictionless regions in aromatic rings, can also generate and stabilize channels in aqueous solutions.

The Hagen–Poiseuille equation is typically employed to estimate the water permeance through 2D nanofiltration membranes per unit area when the fluid behaves as a classical liquid^[^ ^22^ ^]^ which can be represented in the following form:

(1) $$Flux=\frac{h^{4}\times \Delta p}{12L^{2}\times \eta \times \Delta x}$$

where *L* is the average lateral size of the GO flakes, *h* is the interlayer spacing, *η* is the viscosity of water, and Δ*x* is the thickness of the membrane. In our case, with a GO membrane‐based permeation system, the equation can be simplified to:

(2) $$Flux=\frac{1}{L^{2}}$$

Therefore, the obtained membrane, which was stacked with EBI‐nrGO flakes with an average lateral size of 100 nm, experimentally achieved desirable high performance.

## 3 Conclusion

We achieved the self‐assembly of nano‐sized EBI‐nrGO flakes on a substrate with micron‐sized pores. The obtained membranes showed an unprecedented water permeance above 819.1 LMH bar^−1^, with an effective rejection rate of 99.7% for multivalent metal ions. The EBI‐nrGO flakes had relatively pure hydroxyl groups and were prepared by electron beam irradiation and the amino‐hydrothermal method. These hydroxyl groups substantially induce the formation of hydrogen bonds between the EBI‐nrGO flakes in an aqueous solution, coupled with the expansion of the hydrophobic aromatic regions, eventually realizing the stable self‐assembly of the nano‐sized EBI‐nrGO membranes. The obtained membranes exhibited an unprecedented water permeance with an effective rejection rate for multivalent metal ions. Notably, this is the most permeable membrane with a satisfactory rejection rate and outstanding stability for multivalent ions among all state‐of‐the‐art nano‐filtration membranes. Our findings represent a facile step toward achieving ultrahigh permeance, effective rejection, and stable performance for nano‐sized GO membranes for water purification.

## 4 Experimental Section

### Fabrication of EBI‐nrGO Suspension

EBI‐nrGO suspension was prepared by electron beam irradiation (EBI) and amino‐hydrothermal method. GO suspension was prepared from natural graphite powder using a modified Hummers method, using the method described in previous work.^[^ ^17^, ^18^, ^20^ ^]^ The GO suspension (pH 2) was first mixed with isopropanol at a volume ratio of 3:4, then pouched the mixture into a sealed high‐density polyethylene bag after exhausting air with nitrogen, irradiated the mixture with a dose of 40 kGy electron beams produced by a GJ‐II electron accelerator (25 °C, 1.8 MeV and 5 mA).^[^ ^41^ ^]^ Next, 50 mL of the obtained suspension was added to 570 mL DI water and 360 mL NH_4_OH (28%), stirred at 100 °C for 6 h, and further stirred at 90 °C for 1 h. The prepared EBI‐nrGO suspension was used for fabrication of nanofiltration membranes and ion sieving experiments.

### Fabrication of EBI‐nrGO Membranes using Vacuum Filtration

EBI‐nrGO membranes were prepared from EBI‐nrGO suspension using the classical vacuum filtration method.^[^ ^15^, ^18^, ^22^, ^25^, ^42^ ^]^ EBI‐nrGO membranes were prepared from EBI‐nrGO suspension on the mixed cellulose ester (MCE) substrates with a pore size of 0.22 µm using vacuum filtration under 1 bar (Sections S9 and S10; Supporting Information). The effective area of each membrane sample was 11.34 cm^2^ (Figure S7b, Supporting Information). The membrane thickness was tuned by changing the amounts of EBI‐nrGO materials on MCE substrate. Then, the EBI‐nrGO suspensions (12.5 mg L^−1^, 10 mL to 60 mL) were applied for membrane fabrication with the corresponding thickness of 100 to 600 nm.

### Fabrication of EBI‐nrGO Membranes for High Pressure Filtration Experiments

EBI‐nrGO membranes for high pressure filtration experiments were prepared from 12.5 mg L^−1^, 10 mL EBI‐nrGO suspensions on the MCE substrates by employing a nitrogen‐pressurized dead‐end filtration system at 1 bar^[^ ^43^ ^]^ (Section S5; Supporting Information). The effective area of the membrane is 6.06 cm^2^. Then, the FeCl_3_ solutions (200 mL; 50 mg L^−1^) were added at feed side for filtration experiments. A pressure of 1–8 bar was applied by a pressure gas cylinder for filtration experiments (Section S5; Supporting Information).

### Long‐Term Operation Experiments

The long‐term operation experiments of EBI‐nrGO membrane for 50 mg L^−1^ FeCl_3_ were operated by semi‐continuous process. 1000 mL of 50 mg L^−1^ FeCl_3_ was prepared solution in a beaker, and then transported the solution to the feed side with a peristaltic pump, while maintaining ≈300 mL of solution in the feed side, which formed a cyclic flow between the feed side and the beaker.

### Acid‐And‐Alkaline Environment Test

FeCl_3_ solutions (50 mg L^−1^) at pH 3.6 and pH 11.6 were separately added at feed side and filtered at a pressure of 1 bar. During the filtration process, the membranes were still immersed in acid or alkali liquid environments for at least 20 min.

### Theoretical Calculation

The first EBI‐nrGO flake (Figure 3a) was constructed with 120 carbon atoms, three epoxy groups and three hydroxyl groups. The simulation system consists of two EBI‐nrGO flakes with eight water molecules adsorbed. The periodic cubic box has dimensions of 13.5×12.56×35 Å^3^. The second EBI‐nrGO model comprised 120 carbon and two hydroxyl groups, and the corresponding simulation box was also set to 13.5×12.56×35 Å^3^.

The DFT calculations and AIMD simulations were carried out with the CP2K 9.1 packages.^[^ ^44^ ^]^ The global geometry optimization of the systems was first performed and then the eventual optimized systems were applied as the initial structures to run the AIMD simulations. In AIMD simulations, the hybrid Gaussian and plane wave scheme^[^ ^45^ ^]^ in DFT is employed using the Quickstep module. The revised Perdew–Burke–Ernzerh (revPBE)^[^ ^46^ ^]^ exchange‐correlation functional was revised with the DFT‐D3 dispersion correction to obtain a reasonable description of interactions between water and GO sheets.^[^ ^47^ ^]^ The double‐zeta valence polarized basis set was used for all atomic kinds and the electronic cores were represented by Geodecker–Teter–Hutter (GTH) pseudopotentials.^[^ ^48^ ^]^ The electronic density is expanded in the form of plane waves with a cutoff of 460 Ry and the self‐consistent field convergence criterion was chosen as 10^−6^ a.u. The simulation was performed in the canonical ensemble (NVT) and the temperature was maintained at 300 K by a Nosé–Hoover thermostat with a time step of 0.5 fs.

To examine the hydrogen‐bond energy between oxygen functional groups, the crystal orbital Hamilton population (COHP) was performed using the program Local Orbital Basis Suite toward Electronic‐Structure Reconstruction (LOBSTER).^[^ ^49^, ^50^, ^51^ ^]^ Further, the Millikan charge is also obtained through the LOBSTER software package.

### Statistical Analysis

The date presentation as mean ± SD means the average value (mean) and standard deviation (SD) of three independent experiments performed in parallel. And the error bars in figures were plotted in terms of the standard deviations (SDs). Excel and Origin software were used for statistical analysis.

## Conflict of Interest

The authors declare no conflict of interest.

## Author Contributions

F.D. and Z.G. contributed equally to this work. L.C., P.L., and Y.T. conceived the ideas. L.C., Y.T., F.D., Z.G., P.L., L.Y., and S.L. designed the experiments, simulations and co‐wrote the manuscript. F.D., P.L., S.H., B.P., R.Y. and J.J. performed the experiments and prepared the data graphs. Z.G. and Y.T. performed the simulations. All authors discussed the results and commented on the manuscript.

## Supporting information

Supporting Information

Supplemental Movie 1

Supplemental Movie 2

Supplemental Movie 3

Supplemental Movie 4

## Data Availability

The data that support the findings of this study are available in the supplementary material of this article.
