# Supplementary material for: Unexpected Self‐Assembly of Nanographene Oxide Membranes upon Electron Beam Irradiation for Ultrafast Ion Sieving
Source: Adv Sci (Weinh). 2024 Jul 8;11(34):2404001. doi: 10.1002/advs.202404001 (PMC11425961; doi:10.1002/advs.202404001)
Supplement: Supplementary file 1 — Supporting Information [file ADVS-11-2404001-s003.docx]

Supporting Information (SI)

Unexpected Self-assembly of Nanographene Oxide Membranes upon Electron Beam Irradiation for Ultrafast Ion Sieving

Fangfang Dai^1,2,#^, Zonglin Gu^3,#^, Shouyuan Hu^1^, Bingquan Peng^2^, Rujie Yang^4^, Jie Jiang^1^, Lufeng Yao^5^, Shanshan Liang^4^, Yusong Tu^3,^*, Pei Li^1,6,^*, and Liang Chen^1^

*^1^School of Physical Science and Technology, Ningbo University, Ningbo* *315211, China.*

*^2^Wenzhou Institute, University of Chinese Academy of Sciences, Wenzhou, Zhejiang 325000, China.*

*^3^School of Physical Science and Technology & Microelectronics Industry Research Institute, Yangzhou University, Jiangsu 225009, China.*

*^4^Department of Physics, East China University of Science and Technology, Shanghai 200237, China.*

*^5^Department of Basic Courses, Naval University of Engineering, Wuhan 430033, China*

*^6^State Key Laboratory of Surface Physics and Department of Physics, Fudan University, Shanghai 200433, China*

^#^These authors contributed equally: Fangfang Dai, and Zonglin Gu.

*Corresponding author. E-mail: lipei@nbu.edu.cn (P. L.); and ystu@yzu.edu.cn (Y. T.)

**Contents**

[S1 (Section 1): Filtration performance of nanofiltration membranes reported in literatures based on water permeances and rejection rates for multivalent ions. 2](#_Toc169594542)

[S2 (Section 2): Computations of water permeance and rejection rate in filtration experiments. 4](#_Toc169594543)

[S3 (Section 3): The video for high water permeance and rejection of the EBI-nrGO membrane. 5](#_Toc169594544)

[S4 (Section 4): The water permeances and rejection rates of the GO and EBI-nrGO membranes (50 mg/L FeCl_3_).. 6](#_Toc169594545)

[S5 (Section 5): The high-pressure test on the filtration performance of the EBI-nrGO membranes. 7](#_Toc169594546)

[S6 (Section 6): Three periodic filtration organic fouling resistance tests of the EBI-nrGO membranes. 8](#_Toc169594547)

[S7 (Section 7): The video for mechanical property test of the EBI-nrGO membranes. 9](#_Toc169594548)

[S8 (Section 8): Density functional theory calculations for the EBI-nrGO flakes.. 10](#_Toc169594549)

[S9 (Section 9): The fabrication of the EBI-nrGO membranes using vacuum filtration. 11](#_Toc169594550)

[S10 (Section 10): The video for fabrication of the EBI-nrGO membranes using vacuum filtration. 12](#_Toc169594551)

[S11 (Section 11): Characterization methods. 13](#_Toc169594552)

[S12 (Section 12): Concentration variations of FeCl_3_ solutions adsorbed by the EBI-nrGO membranes. 14](#_Toc169594553)

[S13 (Section 13): The surface and cross-sectional SEM images of the GO and EBI-nrGO membranes coating on MCE substrates. 15](#_Toc169594554)

References 14

**S1 (Section 1): Filtration performance of nanofiltration membranes reported in literatures based on water permeances and rejection rates for multivalent ions.**

**Table S1:** Comparisons of different nanofiltration membranes in water permeances and rejection rates for multivalent metal ions.

| Material | Solutions | Water permeance  (LMH bar^−1^) | Rejection rate | Ref |
| --- | --- | --- | --- | --- |
| AH-rGO | FeCl_3_  Pb(NO_3_)_2_ | 43.7  47.4 | 99.96%  99.98% | Ref.^1^ |
| 60℃-GO | FeCl_3_ | 75.0 | 99.9% | Ref.^2^ |
| EBI-rGO | Fe^3+^ | 164.7 | 85.2% | Ref.^3^ |
| PSE-GO-DMF | Cr^3+^ | 13.5 | 91.2% | Ref.^4^ |
| GO&EDA_HPEI 60K | Pb(NO_3_)_2_  NiCl_2_  ZnCl_2_  CdCl_2_ | 5.0 | 95.7%  96%  97.4%  90.5% | Ref.^5^ |
| GO/Torlon hollow fiber membrane | Pd^2+^  Ni^2+^  Zn^2+^ | 4.7 | 95.88%  99.74%  98.07% | Ref.^6^ |
| GO-IPDI membrane | Cr^3+^ | 100.0 | 71.1% | Ref.^7^ |
| GO/TiO2-PDDA | MgCl_2_ | 51.2 | 93.2% | Ref.^8^ |
| GO/QDs | CdCl_2_ | 17.4 | 29.4% | Ref.^9^ |
| GO/PAN | MgCl_2_ | 17.0 | 37.0% | Ref.^10^ |
| PDA/TFC composite membrane | Pb(NO_3_)_2_ | 3.5 | 91.1% | Ref.^11^ |
| TFC membrane on PES support | NiCl_2_ | > 2.0 | 66.0% | Ref.^12^ |
| BPF/PIP-TMC/PES | MgCl_2_ | 11.9 | 49.4% | Ref.^13^ |
| PVA-TMC/PSF | MgCl_2_ | 24.9 | 91.2% | Ref.^14^ |
| MWNT-rGO/PVDF | MgCl_2_ | 11.3 | 9.6% | Ref.^15^ |
| TMV | MgSO_4_ | 62.0 | 98.0% | Ref.^16^ |
| a large-area  graphene-nanomesh/single-walled carbon nanotube (GNM/SWNT) hybrid membrane | Mg^2+^ | 20.6 | 92% | Ref.^17^ |
| Chitosan PES composite membrane | Pb(NO_3_)_3_ | 3.5 | 93.1% | Ref.^18^ |
| EBI-nrGO | **FeCl_3_**  **AlCl**_3_  **CuSO_4_**  **Pb(NO_3_)_2_** | **819.1**  **930.9**  **839.8**  **788.3** | **99.7%**  **95.2%**  **99.7%**  **98.7%** | **This work** |

**S2 (Section 2): Computations of water permeance and rejection rate in filtration experiments.**

After preparing EBI-nrGO membranes by vacuum filtration, 10 to 200 mg L^-1^ of different multivalent metal cations (FeCl_3_, AlCl_3_, CuSO_4_, and Pb(NO_3_)_2_) solutions were added to the feed side, respectively. At a pressure of 1 bar, the salt solutions were filtered through the EBI-nrGO membranes. The water permeance (*J_w_*_)_ was measured by using the following equation (1):

$J_{w}=\frac{V}{\Delta t\times A\times P}$ (1)

where *J_W_* is the water permeance (LMH bar^−1^), *V* is the volume of the filtrate (L), *A* is the effective membrane area (m^2^), ∆*t* is the permeation time (h) and the *P* is the applied pressure (bar).

The rejection rate (*R*) for the multivalent ions was calculated from the concentration of feed and permeate solution. The rejection rate was measured by using the following equation (2):

$R=(1-\frac{C_{P}}{C_{f}})\times100\%$ (2)

where *C_p_* and *C_f_* are the concentration of permeation and feed ions solution which were measured by inductive coupled plasma-optical emission spectrometry (ICP-OES), respectively.

# S3 (Section 3): The video for high water permeance and rejection of the EBI-nrGO membrane.

Please see the video file named ‘Movie_3_5X_in_SI.mp4’.

**Figure S1: Screenshot from the video of filtration experiments.**

#
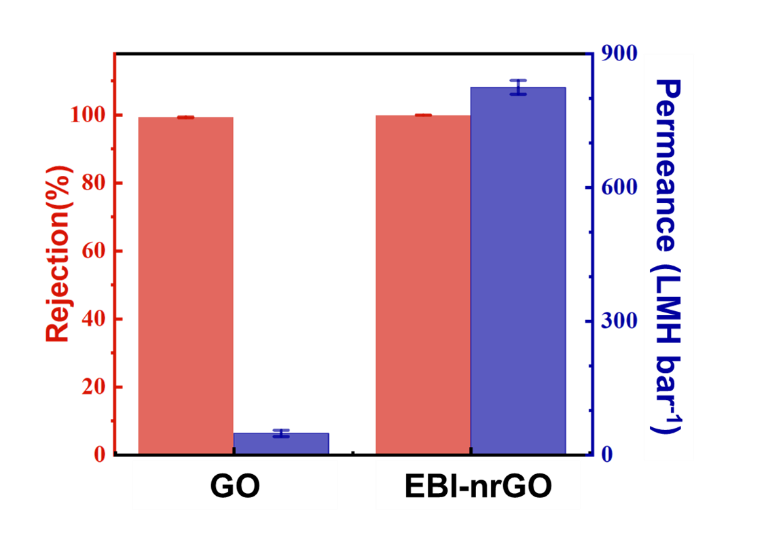
S4 (Section 4): The water permeances and rejection rates of the GO and EBI-nrGO membranes (50 mg/L FeCl_3_).

**Figure S2: The water permeances and rejection rates of the GO and EBI-nrGO membranes (50 mg/L FeCl_3_).**

# S5 (Section 5): The high-pressure test on the filtration performance of the EBI-nrGO membranes.


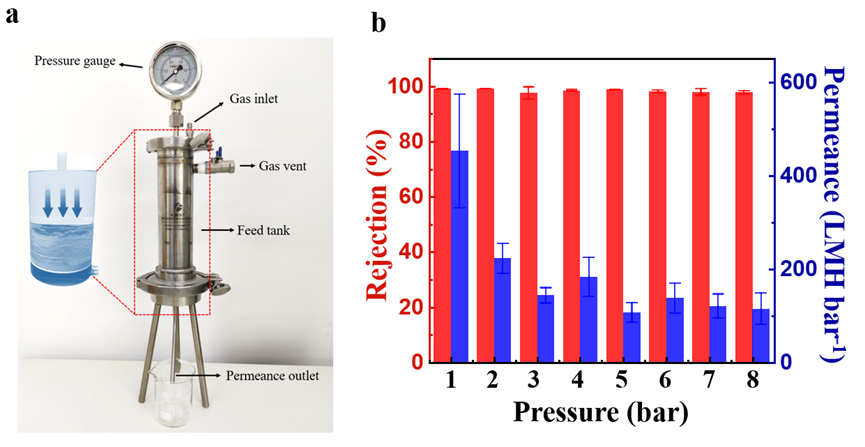


**Figure S3:** **The high-pressure test on the filtration performance of the EBI-nrGO membranes. a** Photo of the pressure filter device. **b** Water permeances and rejection rates of the EBI-nrGO membranes at high pressure in the range of 1-8 bar. Error bars indicate the standard deviation from three different membranes.

**High pressure filtration experiments.** EBI-nrGO membranes for high pressure filtration experiments, were prepared from 12.5 mg L^-1^, 10 mL EBI-nrGO suspensions on the MCE substrates by employing a nitrogen-pressurized dead-end filtration system at 1 bar^19^. The effective area of the membrane is 6.06 cm^2^. Then, FeCl_3_ solutions (200 mL; 50 mg L^-1^) were added at feed side for filtration experiments. A pressure of 1-8 bar was applied by a pressure gas cylinder for filtration experiments. The membranes remain high rejections for FeCl_3_ solution as the pressure increased from 1 to 8 bar. The water permeance decreased and stable at 3 bar. Compared to the vacuum filtration, the pressure filtration exhibited a smaller water permeance, which we attribute to the increased membrane thickness (with the same volume of the EBI-nrGO suspensions loaded on a smaller effective area of MCE substrate) and the decrease in water channels. Therefore, the EBI-nrGO membranes were capable of withstanding a high pressure of 8 bar and maintaining the integrity of the membrane. Such a high mechanical stability is critical for industrial applications.

# S6 (Section 6): Three periodic filtration organic fouling resistance tests of the EBI-nrGO membranes.

**Figure S4:** **Three periodic filtration Organic fouling resistance tests. a** SA solution. **b** HA solution.

**Organic fouling resistance experiments.** Organic fouling resistance experiments were carried out with alternating filtration of pure water and organic pollutant solution, including 200 mg L^-1^ sodium alginate (SA) and humic acid (HA). The complete anti-organic fouling test procedure consisted of three phases and was implemented at 1 bar. Initially, 100 ml of pure water is added to the feed side, and pure water flux in the first 60 mL was integrally recorded. Afterward, organic pollutant solution was used as feed liquid in the second 60 ml. The fouled membrane samples were effectively rinsed and the above operations were repeated until three cycles. In order to estimate the flux recovery rate (FRR), which was obtained by using the following equation:

$FRR=\left( \frac{\boldsymbol{J}_{W1}}{\boldsymbol{J}_{W0}} \right)*100\%$

where ***J***_w0_ is the flux of the membrane for pure water before fouling, and ***J***_w1_ is the membrane flux for water after cleaning.

In the SA fouling test, the flux after fouling decreased sharply compared to the initial water flux in the first filtration cycle as shown in Figure S4a. The permeability declined from 1550.1 to 37.2 LMH bar^-1^. After pure water rinsing, the flux recovered to 1095.1 LMH bar^-1^. After three cycles, the FRR values were 70.6, 84.9 and 94.5%, respectively. For the HA fouling test (Figure S4b), the FRR value were 72.0, 93.5 and 104.6%. All the results suggest that the EBI-nrGO membranes had strong anti-pollution ability to organic pollutants.

# S7 (Section 7): The video for mechanical property test of the EBI-nrGO membranes.

Please see the video file named ‘Movie_2_3X/100X_in_SI.mp4’.

**Figure S5: Screenshot from the video** **of mechanical property test.** GO membrane and EBI-nrGO membrane with a magnetic rotor at 600 rpm for 12 s and 27 min.

#
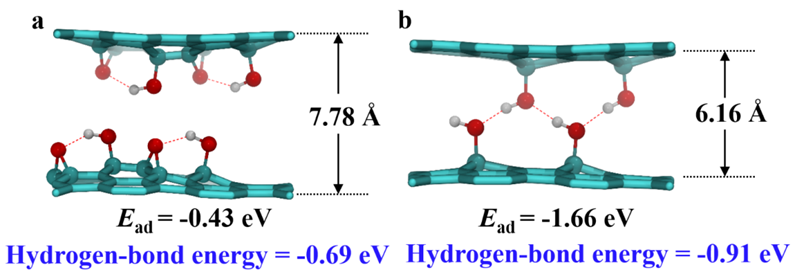
S8 (Section 8): Density functional theory calculations for the EBI-nrGO flakes.

**Figure S6:** **Density functional theory calculations for the GO and EBI-nrGO flakes. a** The optimized structure of two neighboring GO flakes. **b** The optimized structure of two neighboring EBI-nrGO flakes.

For a pair of hydrogen bonds, the AIMD simulations revealed that the hydrogen-bond energy between the hydroxyl groups in two neighboring EBI-nrGO flakes was much stronger than those between the hydroxyl groups in a same plane or between two water molecules in liquid water (Figure 3c).

For the initial GO with numerous oxygen groups, the formation of hydrogen-bond networks is favored due to their close proximity in the same plane. In contrast, the EBI-nrGO, which has a low content of hydroxyl groups and negligible epoxy groups, can weaken the hydrogen-bond network within the plane and strengthen the interactions between the hydroxyl groups among the EBI-nrGO flakes.

To further elucidate this mechanism, we performed calculations on the effect of oxygen content on the interactions between two flakes. We constructed two GO flakes with high oxygen content and two hydroxylated EBI-nrGO flakes, and performed structural optimization using the exchange-correlation function of Perdew–Burke–Ernzerhof (PBE) generalized gradient approximation accompanied by DFT-D3 correction^20,21^. For optimized GO flakes (Figure S6a), hydrogen-bond networks are formed in the plane with a large interlayer spacing, resulting in a small interaction between two GO flakes. However, for optimized EBI-nrGO flakes in Figure S6b, hydrogen bonding is primarily formed by the hydroxyl groups between EBI-nrGO flakes with a relatively small interlayer spacing, resulting in a relatively stronger interaction between them. The adsorption energy between EBI-nrGO flakes was -1.66 eV, which is about four times the adsorption energy of -0.43 eV between GO flakes.

Furthermore, the hydrogen-bond energy in Figure S6a and S6b was -0.69 and -0.91 eV, significantly lower than that between hydroxyl groups in two flakes with only one hydrogen bond (-1.02 eV in Figure 3a). The results suggest that there is a much stronger interactions between the hydroxyl groups among the EBI-nrGO flakes, and a low content of hydroxyl groups can enhance interactions between hydroxyl groups between flakes.

# S9 (Section 9): The fabrication of the EBI-nrGO membranes using vacuum filtration.

**Figure S7: Fabrication of the EBI-nrGO membranes using vacuum filtration. a** Photo of the vacuum filter device. **b** Photo of the obtained the EBI-nrGO membrane. **c** Schematic of the fabrication and water transport channels of the EBI-nrGO membrane.

# S10 (Section 10): The video for fabrication of the EBI-nrGO membranes using vacuum filtration.

Please see the video file named ‘Movie_1_4.5X_in_SI.mp4’.


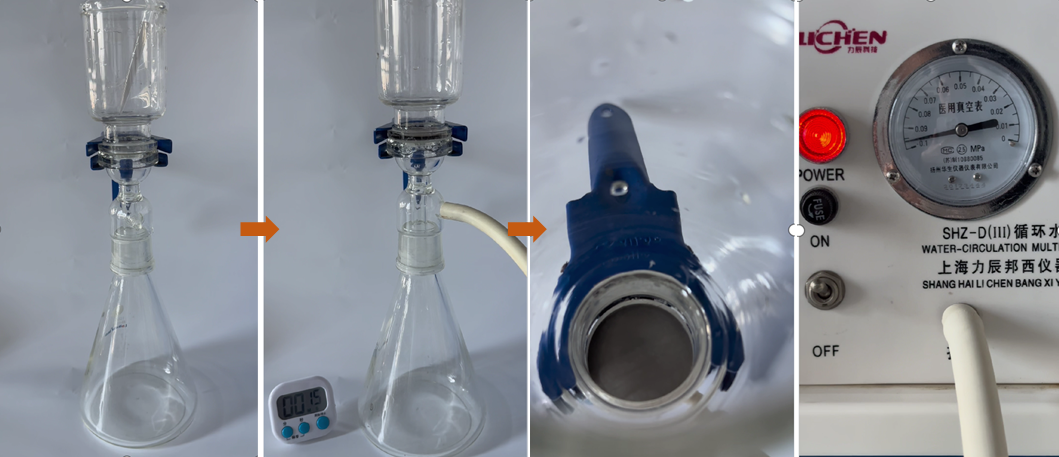


**Figure S8: Screenshot from the video of fabrication of the EBI-nrGO membranes using vacuum filtration.**

# S11 (Section 11): Characterization methods.

The morphology of the EBI-nrGO membranes was characterized by scanning electron microscope (SEM, Hitachi, S-4800) and Transmission electron microscopy (TEM, FEI Talos, F200S). Size distributions of the GO and EBI-nrGO flakes were characterized by BeNano 180 Zeta Pro. X-ray photoelectron spectrometer (XPS) and water contact angles (WCAs) were carried out on Thermo Fisher ESCALAB 250Xi and Biolin Theta. The concentrations of ions solutions were determined using PS7800 inductively coupled plasma optical emission spectrometer (ICP-OES).

# S12 (Section 12): Concentration variations of FeCl_3_ solutions adsorbed by the EBI-nrGO membranes.


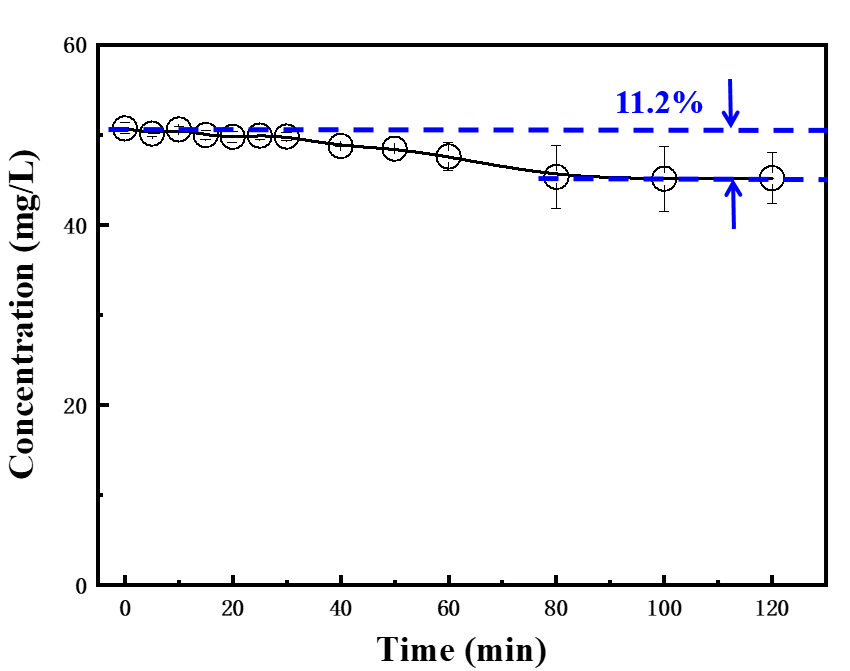


**Figure S9:** **Concentration variations of 50 mg/L FeCl_3_ solutions adsorbed by the EBI-nrGO membranes as a function of adsorption time.** Error bars indicate the standard deviation from three independent parallel experiments.

**Absorption of FeCl_3_ solution by the EBI-nrGO membranes.** We further analyzed the ion adsorption by the EBI-nrGO membranes in our filtration experiments. EBI-nrGO membranes were prepared from the EBI-nrGO suspensions (10 mL; 12.5 mg L^-1^) on substrates using vacuum filtration. Then, FeCl_3_ (200 mL; 50 mg L^-1^) solutions were added at feed side, respectively. These salt solutions were left to rest under ambient conditions (without vacuum filtration). Next, samples were collected at different pre-determined time intervals in 120 min to evaluate the residual salt concentrations of the solutions. We noted that the salt solutions in the feed side permeated very slowly though the EBI-nrGO membranes due to their own gravity, the filtrates were only 5 mL within 120 min. Therefore, the effect of the small volume of filtrates on the salt concentration during the adsorption experiments could be negligible.

The adsorption efficiency (AE) was defined by using the following equation:

$AE=\left( 1-\frac{C_{a}}{C_{0}} \right)*100\%$ (3)

where, *C*o and *C*a is the initial feed concentration and the concentration of salts after adsorption equilibrium, respectively. As shown in Figure S9, the adsorption efficiencies of the EBI-nrGO membranes for FeCl_3_ were approximately 11.2%. Compared to the rejection rates of 93.7-99.8% (Figure 1g), it was much smaller, indicating that the main mechanism of ion removal is the rejection by the EBI-nrGO membranes, although the adsorption effect cannot be neglected.

# S13 (Section 13): The surface and cross-sectional SEM images of the GO and EBI-nrGO membranes coating on MCE substrates.

**Figure S10:** **The surface and cross-sectional SEM images of the GO and EBI-nrGO** **membranes coating on MCE substrates.**

The surface and cross-sectional SEM images show that the surface of the EBI-nrGO membranes is continuous, without any macroscopic pores and defects, and the thickness of the membranes measured from the cross-sectional SEM images is approximately 100 nm. **References**

1. F. Dai, R. Yu, R. Yi, J. Lan, R. Yang, Z. Wang, J. Chen, L. Chen, *Chemical Communications*, **2020**, *56*, 15068-15071.

2. F. Dai, F. Zhou, J. Chen, S. Liang, L. Chen, H. Fang, *Journal of Materials Chemistry A*, **2021**, *9*, 10672-10677.

3. R. Yi, X. Xia, R. Yang, R. Yu, F. Dai, J. Chen, W. Liu, M. Wu, J. Xu, L. Chen, *Carbon*, **2021**, *172*, 228-235.

4. R. Mukherjee, P. Bhunia, S. De, *Chemical Engineering Journal*, **2016**, *292*, 284-297.

5. Y. Zhang, S. Zhang, T. S. Chung, *Environmental science & technology*, **2015**, *49*, 10235-10242.

6. Y. Zhang, S. Zhang, J. Gao, T. S. Chung, *Journal of membrane science*, **2016**, *515*, 230-237.

7. P. Zhang, J. L. Gong, G. M. Zeng, C. H. Deng, H. C. Yang, H. Y. Liu, S. Y. Huan, *Chemical Engineering Journal*, **2017**, *322*, 657-666.

8. M. Zhang, K. Guan, Y. Ji, G. Liu, W. Jin, N. Xu, *Nature communications*, **2019**, *10*, 1-8.

9. G. Zhao, R. Hu, X. Zhao, Y. He, H. Zhu, *Journal of Membrane Science*, **2019**, *585*, 29-37.

10. M. Zhang, J. Sun, Y. Mao, G. Liu, W. Jin, *Journal of Membrane Science*, **2019**, *574*, 196-204.

11. T. Wang, J. Bai, P. Li, B. Wang, L. Du, G. Tao, *High Energy Density Physics*, **2016**, *19*, 65-75.

12. H. Mahdavi, F. Razmi, T. Shahalizade, *Separation and Purification Technology*, **2016**, *162*, 37-44.

13. Y. J. Tang, Z. L. Xu, S. M. Xue, Y. M. Wei, H. Yang, *Journal of Membrane Science*, **2017**, *541*, 483-491.

14. Z. Tan, S. Chen, X. Peng, L. Zhang, C. Gao, *Science*, **2018**, *360*, 518-521.

15. Y. Han, Y. Jiang, C. Gao, *ACS applied materials & interfaces*, **2015**, *7*, 8147-8155.

16. L. Gui, J. Dong, W. Fang, S. Zhang, K. Zhou, Y. Zhu, Y. Zhang, J. Jin, *Nano Letters*, **2020**, *20*, 5821-5829.

17. Y. Yang, X. Yang, L. Liang, Y. Gao, H. Cheng, X. Li, M. Zou, R. Ma, Q. Yuan, X. Duan, *Science*, **2019**, *364*, 1057-1062.

18. S. Zhang, M. H. Peh, Z. Thong, T. S. Chung, *Industrial & Engineering Chemistry Research*, **2015**, *54*, 472-479.

19. R. Yang, Y. Fan, R. Yu, F. Dai, J. Lan, Z. Wang, J. Chen and L. Chen, *Journal of Membrane Science*, **2021**, *635*, 119437.

20. J. Klimeš, D. R. Bowler and A. Michaelides, *Physical Review B*, **2011**, *83*, 195131.

21. J. P. Perdew, K. Burke and M. Ernzerhof, *Physical review letters*, **1996**, *77*, 3865.
